# Assessment of haemostasis in pregnant women: A retrospective evaluation of the diagnostic performance of the HEMSTOP standardised questionnaire

**DOI:** 10.1097/EA9.0000000000000050

**Published:** 2024-03-15

**Authors:** Tamara Zec, Denis Schmartz, Pomeline Temmerman, Jean-François Fils, Brigitte Ickx, Fanny Bonhomme, Philippe Van Der Linden

**Affiliations:** From the Brugmann University Hospital, Brussels, Université Libre de Bruxelles, Belgium (TZ, DS, VDL), Université libre de Bruxelles, Belgium (PT), Erasme University Hospital, Brussels, Belgium (BI), Ars Statistica, Nivelles, Belgium (JFF), Geneva University Hospital, Switzerland (FB)

## Abstract

**BACKGROUND:**

Contemporary guidelines pertaining to the evaluation of bleeding risk recommend conducting a comprehensive examination of both personal and family histories concerning haemorrhagic diatheses.

**OBJECTIVES:**

We employed the standardised HEMSTOP (Hematoma, hEmorrhage, Menorrhagia, Surgery, Tooth extraction, Obstetrics, Parents) questionnaire in pregnant women to evaluate its efficacy in detecting a haemostatic disorder and predicting the risk of haemorrhage associated with delivery.

**DESIGN:**

A single-centre retrospective observational cohort study.

**SETTING:**

Brugmann Hospital, a tertiary university institution.

**PATIENTS:**

All full-term parturients who underwent vaginal or caesarean delivery in our hospital between January 2020 and December 2021 were included in the study. A total of 3588 patients were enrolled.

**MAIN OUTCOME MEASURES:**

The primary aim of this study was to assess the sensitivity and specificity of the HEMSTOP questionnaire in identifying individuals with an abnormal primary haemostatic profile. The secondary objective was to evaluate the sensitivity and specificity of the HEMSTOP questionnaire in predicting postpartum haemorrhage (PPH; defined as blood loss >1000 ml). Additionally, positive-predictive values and negative-predictive values (NPVs) were calculated.

**RESULTS:**

The specificity and sensitivity of the HEMSTOP questionnaire to predict an abnormal standard coagulation test in pregnant women are respectively 96% [95% confidence interval (CI), 0.95 to 0.97] and 39% (95% CI, 0.20 to 0.61). Its NPV is 100%. The specificity and sensitivity of the HEMSTOP questionnaire to predict postpartum bleeding risk are respectively 96% (95% CI, 0.95 to 0.97) and 8% (95% CI, 0.06 to 0.11).

**CONCLUSION:**

In the conditions of our study, the HEMSTOP questionnaire enables the prediction of a primary haemostatic anomaly with a specificity and sensitivity comparable to routine haemostatic assessments. These findings concur with the recommendation against the routine prescription of laboratory tests for patients lacking a history of bleeding diathesis.

**TRIAL REGISTRATION:**

Clinical Trial NCT 05191251.

**KEY POINTS**

- In pregnant women, a standardised HEMSTOP questionnaire with a score less than 2 is associated with a sensitivity of 39%, a specificity of 96% and a NPV of 100% of having an abnormal coagulation tests.
- A HEMSTOP questionnaire with a score less than 2 is associated with a sensitivity of 8%, a specificity of 96% and a NPV of 89% of having postpartum bleeding.

## Introduction

Bleeding associated with any surgical or obstetric intervention can adversely affect the clinical outcome of the patient.^1–3^ Therefore, pre-interventional haemostatic assessment represents an essential step to minimise the risk of bleeding. This assessment begins with a detailed survey of the personal as well as the family history, and a meticulous clinical examination.^1,2^

Severe maternal haemorrhage is defined as a cumulative blood loss at least 1000 ml or blood loss accompanied by signs and symptoms of hypovolaemia within 24 h of delivery.^4–6^ It is a major risk factor for maternal morbidity and mortality that can lead to disseminated intravascular coagulopathy, emergency hysterectomy and haemorrhagic shock resulting in multiorgan failure.^4,7–9^ Although maternal mortality from haemorrhage has decreased significantly over the past decade, postpartum haemorrhage (PPH) remains the leading cause of death associated with delivery, varying between 9.3% in countries with a high socio-economic level and 45.7% in countries with a low socio-economic level.^6,7,9–11^

The relationship between standard biological haemostatic tests and the risk of bleeding is quite complex. Normal standard biological results do not exclude any haemorrhagic risk related to the patient. Conversely, in many situations, abnormal standard biological tests do not accurately predict the bleeding risk during an invasive procedure.^1,3,12^ As a consequence, current guidelines recommended first the evaluation of haemorrhagic risk from a personal and family history, specifically oriented to the risk of a haemorrhagic diathesis and to order a biological haemostatic assessment only in patients whose clinical history and examination lead to the suspicion of a haemostatic disorder, regardless of the patient's age, the ASA grade, the type of intervention and the type of anaesthesia chosen (general anaesthesia, neuraxial anaesthesia, peripheral blocks or combined techniques).^1^

However, only an interview conducted in a structured manner will have a good predictive value, underlining the relevance of setting up a standardised, simple, easy and quick screening questionnaire to apply in anaesthesia consultations.^3^ The HEMSTOP (Hematoma, hEmorrhage, Menorrhagia, Surgery, Tooth extraction, Obstetrics, Parents) questionnaire developed by Fanny Bonhomme *et al.*^2^ and used in our daily clinical practice during anaesthesia consultations meets these reqirements.^3^ It has been prospectively evaluated and validated in the HEMORISQ study, which involved 1405 patients who underwent different types of surgery, excluding cardiac, neurological, vascular, thoracic and obstetric.^13,14^ The results showed that the diagnostic performance of the structured questionnaire on bleeding risk was acceptable but only in women. Of note, of the seven questions in the HEMSTOP questionnaire, two are specific to the female population.^14^

The aim of the present retrospective study was to determine the diagnostic performance of the standardised HEMSTOP questionnaire in the obstetric population when completed during our routine preprocedure anaesthesia consultation in our institution. The primary objective of the study was to evaluate the sensitivity and specificity of the HEMSTOP questionnaire in identifying patients with an abnormal primary haemostatic profile, who will require a further evaluation with specific laboratory tests and/or with a dedicated consultation with a haematologist. The secondary objective of the study was to evaluate the sensitivity and the specificity of the HEMSTOP questionnaire in predicting PPH.

## Materials and methods

Brugmann hospital is a tertiary university institution, with about 3000 deliveries per year in its maternity unit. A consultation is organised by the department of anaesthesia for any parturient scheduled for a caesarean section or whose delivery is planned vaginally with epidural analgesia. For emergency cases and those requesting an epidural during labour, an immediate anaesthetic consultation is undertaken.

During this consultation, the HEMSTOP questionnaire (Table 1) is systematically carried out.^2^ This simple and unambiguous questionnaire is made up of seven questions, two of which are specific to women.^2^ The patients answer the questions in a binary way. Each positive response is scored ‘1’ and each negative response ‘0’.^2^ The questionnaire is said to be positive when the score is greater than or equal to 2.^2^

**Table 1:** HEMSTOP questionnaire

Q1	Have you ever consulted a doctor or received treatment for prolonged or unusual bleeding (such as nosebleeds, minor wounds) ?
Q2	Do you experience bruises/hematomas larger than 2 cm without trauma or severe bruising after minor trauma
Q3	After a tooth extraction, have you ever experienced prolonged bleeding requiring medical/dental consultation ?
Q4	Have you experienced excessive bleeding during or after surgery?
Q5	Is there anyone in your family who suffers from a coagulation disease (such as haemophilia, von Willebrand disease, etc.)?^a^
Q6A	Have you ever consulted a doctor or received a treatment for heavy or prolonged menstrual periods (contraception pill, iron, etc.)?
Q6B	Did you experience prolonged or excessive bleeding after delivery ?

Data from Ref.^2^. HEMSTOP, Hematoma, Haemorrhage, Menorrhagia, Surgery, Tooth extraction, Obstetrics, Parents. Questions Q6A and Q6B are questions for female patients.

a This question related to family members and not to the patient.

During the consultation, standard biological haemostatic tests are also requested: prothrombin time (PT), international normalised ratio (INR), activated partial thromboplastin time (aPTT), fibrinogen level and platelet count. Patients can have these tests undertaken the laboratory of their choice. A PT less than 70%, an INR greater than 1.2, a plasma fibrinogen level lower than 1.5 g l^−1^, and a platelet count less than 75 000 × 10^9^  l^−1^ were considered as abnormal values. For the aPTT, abnormal values were defined according with the ranges applied in the different laboratories having performed the test.

In the presence of an abnormal aPTT test, second-line haemostatic tests included the determination of von Willebrand factor (vWF) antigen and activity, FVIII, FIX, FXI and platelet function analysis in the presence of adenosine diphosphate as an agonist (PFA-ADP) test.

In case of an abnormal PT value, second-line haemostatic tests include the determination of FII, FVII, FX and FV. In cases where oral administration of vitamin K did not normalise the PT value, a consultation with a haematologist is organised. A consultation with the haematologist is also organised in the event of low plasma fibrinogen and/or low platelet count to determine the aetiology of the abnormal result.

Peripartum blood loss was determined by measuring blood collected in bags and intra-operative suction cannisters, and by weighing surgical swabs.^6,15^ PPH was defined as a blood loss at least 1000 ml.^6,15^

Ethical approval for this study (Ethical Committee 2021/189) was provided by the Ethical Committee of the Brugmann University Hospital, Brussel, Belgium on 16 November 2021.

### Data collection

Following the agreement of the ethics committee (CE 2021/189) of the Brugmann University Hospital, the medical records of all full-term parturients who gave birth vaginally or by operative delivery in the maternity ward of our institution from 01 January 2020 to 31 December 2021 were reviewed. The study protocol was recorded on Clinical.Trial.org (NCT 05191251) before patient inclusion.

#### Inclusion criteria

1. Parturients for whom a HEMSTOP questionnaire had been carried out prepartum, during the predelivery anaesthesia consultation
2. Age 18 years and older

#### Exclusion criteria

1. Patient under the age of 18
2. Patients taking long-term anticoagulants and/or anti-aggregants
3. Patients with a language barrier

Data collected were:

1. Standardised HEMSTOP questionnaire as it is usually carried out by the anaesthesiologists during the predelivery consultation and recorded in the electronic patient record (XCare, Xperthis, Brussels, Belgium)
2. Results of routine haemostatic tests: PT, INR, aPTT, fibrinogen level and platelet count
3. The results of second-line haemostatic tests evaluating the intrinsic and extrinsic pathways of coagulation, and platelet function, if applicable
4. Peripartum blood loss
5. Prepartum and postpartum haemoglobin levels

### Statistical analysis

For continuous data, we tested the assumptions of the *t* test: determine if groups have comparable variances with Bartlett's test for homogeneity of variance and if the residuals of the *t* test are normally distributed. When one of the underlying assumptions was not met, a nonparametric approach was used with Wilcoxon-signed-rank test. Categorical variables were analysed with a χ-square test. Data are presented as mean ± SD, medians [IQR] and *n* (%) as appropriate. Specificities, sensitivities, positive-predictive values and NPVs were calculated with the **epiR** package.

The R software (R Core Team, 2021), version 4.2.0. was used to analyse the data.

## Results

**FIGURE 1:**
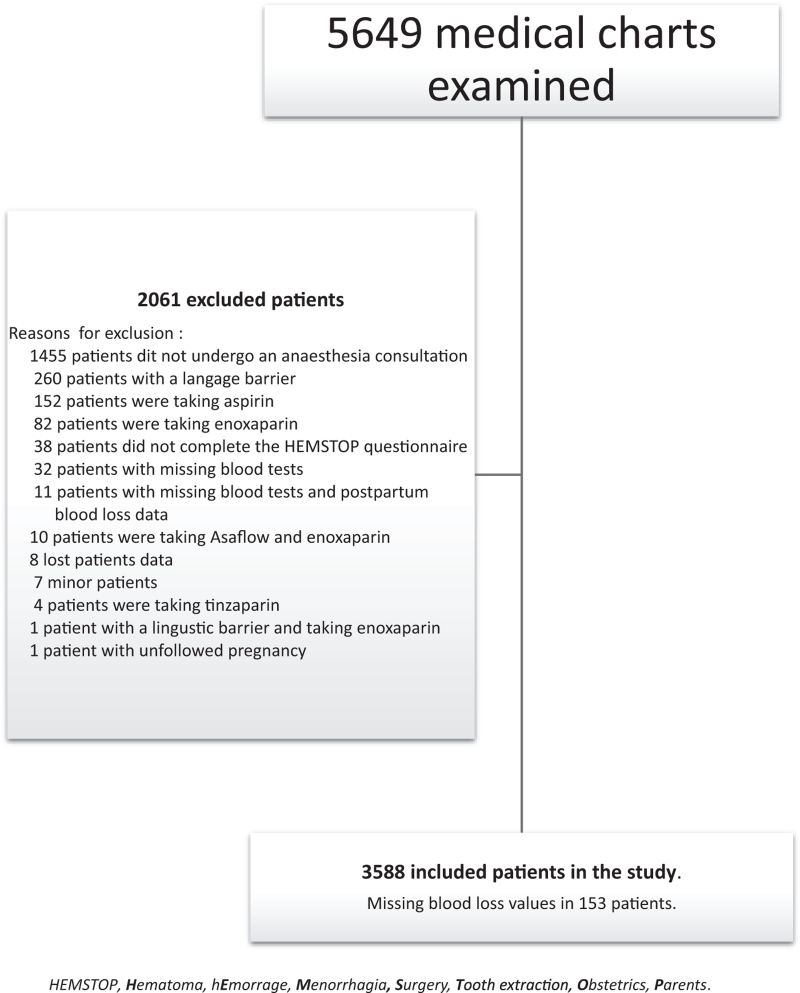
Flow chart for the study.

During the study period, 5649 patients gave birth in the maternity ward of our institution. From these, 3588 were included in the study (Fig. 1).

**FIGURE 2:**
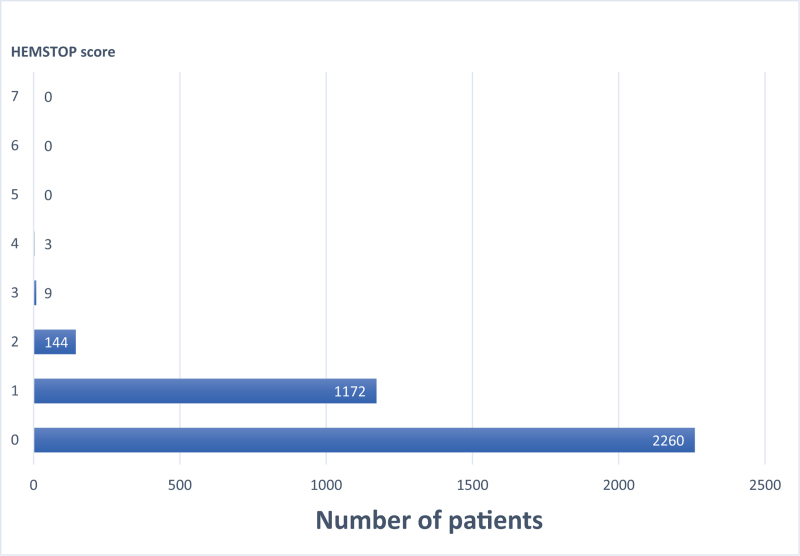
The distribution of patients based on the HEMSTOP score.

Patients’ characteristics are shown in Table 2. The distribution of patients based on the HEMSTOP score is depicted in Fig. 2.

**Table 2:** Demographic characteristics

Patients included (3588)	
Age (years)	30 [26 to 34]
Weight before pregnancy (kg)	65 [58 to 76]
Weight at childbirth (kg)	77 [69 to 88]
Height (cm)	164 [160 to 168]
BMI before the pregnancy (kg m^−2^)	24.4 [21.4 to 28.2]
BMI at childbirth (kg m^−2^)	28.7 [25.7 to 32.5]
Gestational age (weeks)	39 [38 to 40]
ASA score	
I	879 (25)
II	2664 (75)
III	45 (1)
IV	0 (0)
Primiparous	1099 (31)
Vaginal delivery	2907 (81)
Caesarean delivery	681 (19)

Data are presented as median [IQR], and *n* (%).

The HEMSTOP questionnaire is considered positive when the score is equal to or > 2. HEMSTOP, Hematoma, hEmorrage, Menorrhagia, Surgery, Tooth extraction, Obstetrics, Parents.

In our study population, 156 (4.3%) parturients had a positive HEMSTOP questionnaire. The question with the most frequently positive answer was question 6A (menorrhagia, 36.2%, Table 3). Among these, 99% received iron treatment and no one received oral contraception or tranexamic acid for heavy or prolonged menstrual periods. The second most frequently positive answer refers to question 6B (prolonged or excessive bleeding after delivery, 4.4%, Table 3).

**Table 3:** Qualitative analysis of symptoms among patients

Positive symptoms (according to the questionnaire)	
Q1 (prolonged/unusual bleeding)	31 (0.86)
Q2 (bruises/haematomas)	2 (0.06)
Q3 (bleeding after tooth extraction)	1 (0.028)
Q4 (postoperative bleeding)	6 (0.17)
Q5 (haemostasis disorders in a family member)	4 (0.11)
Q6A (menorrhagia)	1298 (36.2)
Q6B (prolonged or excessive bleeding after delivery)	157 (4.4)
HEMSTOP score >2	156 (4.3)
Abnormal primary haemostasis assessment	
Prolonged PT (<70%)	0 (0)
Prolonged aPTT (s)	23 (0.64)
Abnormal platelet count (75 000 × 10^9^ l^−1^)	3 (0.08)
Plasma fibrinogen level <150 mg dl^−1^	0 (0)
Abnormal secondary haemostatic assessment	
PFA-ADP	3 (0.08)
Coagulation factor assay	21 (0.58)
Bleeding disorder (patient)s	
Known before pregnancy	8 (0.22)
Unknown before pregnancy	4 (0.11)
Acquired (severe sepsis)	2 (0.06)
Known bleeding disorders	
Type 1 Von Willebrand severe	1 (0.03)
Factor V deficiency	1 (0.03)
Factor VII deficiency	2 (0.56)
Factor XI deficiency	3 (0.08)
Factor XII deficiency	1 (0.03)
Discovered haemostatic disorders	
Factor XI deficiency	4 (0.11)
Other factors deficiency	0 (0)

Data are presented as *n* (%).

In our study population, 23 had abnormal haemostatic screening; these were all related to a prolonged aPTT (Table 3). No patient had an abnormal PT or a low plasma fibrinogen level (Table 3). Three patients with a prolonged aPTT exhibited a low platelet count: the secondary assessment diagnosed this as pregnancy-induced thrombocytopenia (Table 3).

**FIGURE 3:**
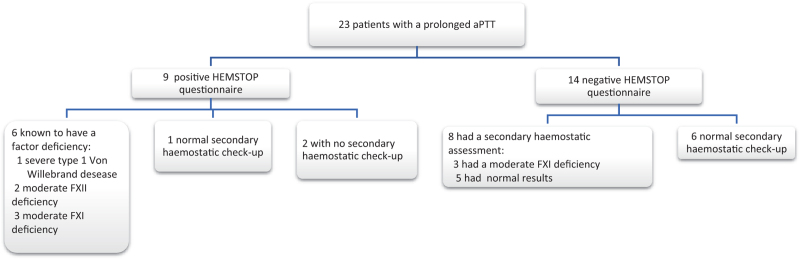
Flow chart for patients with a prolonged activated partial thromboplastin time.

Among the 23 patients with a prolonged aPTT (Table 3), 6 patients were known to have a clotting factor deficiency, 6 had a normal secondary haemostatic check-up, 6 did not have a secondary haemostatic check-up, 3 were diagnosed with factor XI deficiency and 2 had a prolonged aPTT in a context of severe sepsis from chorioamnionitis (acquired bleeding disorder). Nine of these 23 patients had a positive HEMSTOP questionnaire. Of these, 6 patients were known to have a clotting factor deficiency (1 patient with severe type 1 Von Willebrand disease, 2 patients with moderate FXII deficiency and 3 patients with moderate FXI deficiency). Among the 3 other patients with a positive HEMSTOP questionnaire, 1 patient had a normal secondary haemostatic check-up and 2 patients did not have a secondary haemostatic check-up (Fig. 3). Fourteen of these 23 patients had a negative HEMSTOP questionnaire. A secondary haemostatic assessment was undertaken in eight of them. Of these, three patients were diagnosed with moderate FXI deficiency. In the other five patients, the secondary haemostasis assessment found aPTT values in the normal range (Fig. 3).

aPTT, activated partial thromboplastin time; HEMSTOP, Hematoma, hEmorrage, Menorrhagia, Surgery, Tooth extraction, Obstetrics, Parents; factor XI; FXII, factor XII.

Among the eight patients who reported having a clotting factor deficiency (Table 3), all had a positive HEMSTOP questionnaire, although two of them had a normal aPTT.

Of the four patients not known to have a clotting deficiency before pregnancy (Table 3), three patients had a positive HEMSTOP questionnaire. Of these three patients, two patients also had a prolonged aPTT. The only patient with a negative HEMSTOP questionnaire, had a prolonged aPTT, which justified a secondary haemostatic check-up. In these four patients, a moderate FXI deficiency was diagnosed through this secondary haemostatic assessment. As a preventive measure, these four patients received 1 g of tranexamic acid before delivery, and only one patient experienced PPHe.

A significant relationship was observed between the result of the HEMSTOP questionnaire and the presence of abnormal standard coagulation tests (Table 4).

**Table 4:** Relationship between the primary haemostatic assessment and the HEMSTOP questionnaire

	Normal	Abnormal
	primary haemostatic assessment	primary haemostatic assessment
Negative questionnaire HEMSTOP	3418 (99.62%)	14 (0.41%)
Positive questionnaire HEMSTOP	147 (94.23%)	9 (5.77%)

The χ-squared test indicates (*χ*^2^ = 17.056, *df* = 1, *P* value <0.001). The sensitivity (true positive rate) equals 0.39 (95% CI, 0.20 to 0.61), and the specificity (true negative rate) equals 0.96 (95% CI, 0.95 to 0.97). The positive-predictive value equals 0.06 (95% CI, 0.03 to 0.11) and the negative-predictive value equals 1.00 (95% CI, 0.99 to 1.00).

A significant relationship exists between the HEMSTOP questionnaire and the presence of PPH (Table 5).

**Table 5:** Relationship between postpartum haemorrhage and the HEMSTOP questionnaire

	Normal bleeding	Abnormal bleeding
Negative HEMSTOP questionnaire	2926 (89.18%)	355 (10.82%)
Positive HEMSTOP questionnaire	122 (79.22%)	32 (20.78%)

The χ-squared test indicates (*χ*^2^ = 13.615, *df* = 1, *P* value <0.001). The sensitivity (true positive rate) equals 0.08 (95% CI, 0.06 to 0.11), and the specificity (true negative rate) equals 0.96 (95% CI, 0.95 to 0.97). The positive-predictive value equals 0.21 (95% CI, 0.15 to 0.28) and the negative-predictive value equals 0.89 (95% CI, 0.88 to 0.90).

Compared with patients with a negative HEMSTOP questionnaire, those with a positive one had a lower predelivery haemoglobin, platelet count, PT and fibrinogen and a higher predelivery aPTT. They exhibited also a higher postpartum blood loss (Table 6).

**Table 6:** Comparison of the variables analysed in our study population between the group of patients with positive HEMSTOP score and the group of patients with negative HEMSTOP score

Variables	Negative HEMSTOP questionnaire	Positive HEMSTOP questionnaire	*P* value
Predelivery haemoglobin (g dl^−1^)	11.9 [11.1 to 12.6]	11.5 [10.7 to 12.2]	<0.001
Predelivery platelets (10^3^ μl^−1^)	215 [178 to 257]	206 [168 to 248]	0.01265
Predelivery PT (%)	120 [112 to 131]	117 [108 to 127]	0.00572
Predelivery aPTT (s)	24.5 [23.3 to 25.9]	25.2 [23.8 to 26.8]	<0.001
Predelivery fibrinogen (mg dl^−1^)	474 [426 to 520]	445 [406 to 501]	0.00206
Postpartum blood loss (ml)	300 [160 to 550]	400 [200 to 858]	<0.001

Data are presented as median [IQR]. aPTT, activated partial thromboplastin time; PT, prothrombin time.

## Discussion

The objective of this retrospective study was to assess the diagnostic performance of the HEMSTOP questionnaire in detecting coagulation abnormalities in full-term pregnant women. For this purpose, we used the simple and standardised HEMSTOP questionnaire developed by Bonhomme *et al.*^2^ In their validation study, Bonhomme *et al.* reported a specificity of 98.6% and sensitivity of 89.5% for the HEMSTOP questionnaire in detecting haemostasis disorders with haemorrhagic risk.^2^ In the context of our study, the standardised HEMSTOP questionnaire exhibited robust predictive capabilities for a negative haemostatic assessment, with a 100% NPV, 96% specificity and 39% sensitivity.

With respect to the specificity of the HEMSTOP questionnaire, our findings align with those reported by Bonhomme *et al.*^2^ and support current recommendations advising against the routine request for primary haemostatic tests for all individuals. in addition, our results underscore the utility of a standardised questionnaire for screening patients who exhibit a personal/family history or clinical signs indicative of a coagulation anomaly.^1^

The rationale for investigating coagulation abnormalities in pregnant women stems from the risks of PPH and complications such as haematoma during the administration of epidural or spinal anaesthesia. In our retrospective study of patient samples, no cases of spinal haematoma complications were documented.

With regards to the sensitivity of the HEMSTOP questionnaire, our results are less favourable compared with those reported by Bonhomme *et al.* (39 versus 89.5%). This disparity could be attributed to differences in the methodologies employed in the two studies. In their validation study, Bonhomme *et al.*^2^ included a small number of healthy patients along with retrospectively selected patients with either a confirmed coagulation disorder or a positive bleeding diathesis. In our study, we assessed a general population of pregnant women, where the prevalence of hereditary coagulation disorders and platelet dysfunction is known to be very low. The prevalence of coagulation abnormalities observed in our study population aligns with that documented in the literature.^1,3^ Indeed, various studies indicate that the prevalence of coagulation abnormalities in the healthy general population ranges from 0.5 to 16% when considering all primary haemostatic tests, including PT, aPTT and platelet count.^1^ The extremely low prevalence of haemostasis anomalies in our population contributes to the very high NPV of the HEMSTOP questionnaire in predicting a haemostatic abnormality. One-third of patients with prolonged aPTT did not undergo a secondary haemostatic examination, representing a bias contributing to the reduced sensitivity of the analysis.

Table 6 elucidates the importance of the HEMSTOP questionnaire. Despite the questionnaire's low sensitivity, a comprehensive personal and familial medical history proves adept at alerting one to haemostatic impairments and enhancing team responsiveness in anticipation of haemorrhagic events.

In the context of our study, the HEMSTOP questionnaire predicts a bleeding risk in pregnant women with a sensitivity of 8% and a specificity of 96%. These results are largely comparable with those obtained with standard coagulation tests.^1^ Two studies, one in 1985 by Kaplan *et al.*^16^ and the other in 1995 by Perez *et al.*^17^ demonstrated that the systematic performance of primary haemostasis assessments in all patients has minimal impact on modifying patient care. More recently, a French epidemiological study reported that the appropriate management of patients classified at risk based on abnormal standard haemostatic tests, was followed in less than 15% of cases, with only 0.57% receiving corrective treatment.^1^ Given the low diagnostic performance and limited therapeutic impact of primary haemostatic assessments, several international scientific societies (American Society of Anesthesiologists, British Committee for Standards in Haematology recommend against routine requests for systematic haemostatic tests to assess the risk of bleeding before surgery or invasive procedures, including neuraxial anaesthesia.^1,18^ To date, only the Italian Society for Hemostasis and Thrombosis (SISET) recommends a systematic primary haemostasis assessment in these situations, justifying this by the low additional cost incurred.^19^

In our study population, 36% of patients responded affirmatively to the question regarding menorrhagia, consistent with the prevailing literature reporting a prevalence of menorrhagia ranging from 18 to 38% in women of childbearing age.^20^ Remarkably, in our study cohort, 99% of patients reported having received iron as a treatment for menorrhagia.

Four percentage of the patients included in the study reported a history of ‘prolonged or excessive bleeding after delivery’, in contrast to the prevalence rates of PPH ranging from 1.2 to 1.25% reported in the literature.^15^ This difference could likely be attributed to the lack of consensus regarding the definition of PPH, which varies from country to country, as well as the diverse and imprecise quantification methods used for PPH. Furthermore, question 6B of the HEMSTOP questionnaire, which refers to ‘prolonged or excessive bleeding after delivery’, appears to be an imprecise question: ‘prolonged or excessive bleeding after delivery’ as stated by the patient may not meet the requirements to be classified as PPH.

In our population, four patients were diagnosed peripartum with a moderate factor XI deficiency based on a positive HEMSTOP questionnaire and/or a prolonged aPTT. During a normal pregnancy, the levels of several coagulation factors increase gradually until the third trimester (factors VII, VIII, X, XII, fibrinogen and Von Willebrand), whereas others increase only slightly or not at all (factors II, V, IX, XI and XIII).^21,22^ The moderate factor XI deficiencies identified in our patients may not be associated with an increased bleeding risk that requires specific treatment or prevention.

Our study presents several limitations. Firstly, there is a potential for selection bias owing to the retrospective nature of our analysis. Secondly, in our centre, it is routine practice to conduct primary haemostatic assessments for all patients before childbirth, and therefore, a secondary haemostatic assessment is not consistently performed for all our patients. Thirdly, we did not include patients undergoing treatment with anticoagulants and/or antiplatelet agents, preventing the evaluation of the diagnostic performance of the HEMSTOP questionnaire in this specific population. Fourthly, the questionnaire is formulated and validated in French; consequently, our findings may not be extrapolated to patients who do not speak this language. Patients with a language barrier were excluded from the study. Additionally, we did not include the consideration of risk factors for PPH in the analysis of our population, which may introduce a confounding bias. This aspect will be addressed in a subsequent study. Finally, the questionnaires were not consistently administered by consultant anaesthesiologists. Nevertheless, when residents conducted the consultation, they were under the supervision of consultants.

Our study also has several strengths. To the best of our knowledge, this is the first study to assess the diagnostic performance of the HEMSTOP questionnaire in a population of pregnant women. The evaluated population constituted a large number of parturients under our care in the maternity ward of tertiary university hospital, with very few exclusion criteria, thereby enhancing the generalisability of our results. The validation study of the HEMSTOP questionnaire in the obstetric population is currently underway.

## Conclusion

In the conditions of our study, the HEMSTOP questionnaire demonstrated a sensitivity and specificity comparable to primary haemostatic tests in predicting the haemorrhagic risk within our studied population of pregnant women. These results align with current recommendations against routine requests for laboratory coagulation tests in patients with no history of a bleeding diathesis. A validation study of the HEMSTOP questionnaire and a prospective evaluation within the general population would provide an opportunity to refine its diagnostic performance. The ongoing French multicentre HEMORISQ study, involving a large cohort of patients undergoing scheduled surgery, is currently finished, with results pending.
